# Osteochondroma of the Rib: A Potentially Life-Threatening Benign Tumor

**DOI:** 10.7759/cureus.45449

**Published:** 2023-09-18

**Authors:** Laura C Morales, Jose D Cardona Ortegón, Bibiana A Pinzón Valderrama, Ana M Jiménez Uribe, Nicolas G Mora Bendeck, Fernando Fierro Ávila

**Affiliations:** 1 Radiology, University Hospital Fundación Santa Fe de Bogotá, Bogotá, COL; 2 Radiology, University Hospital Fundación Santa Fé de Bogotá, Bogotá, COL; 3 Pediatric Surgery, University Hospital Fundación Santa Fe de Bogotá, Bogotá, COL

**Keywords:** surgery, hemothorax, emergency room, tumor, rib, osteochondroma

## Abstract

Osteochondroma is the most common benign bone tumor. It can be classified as isolated or multiple. While the majority of osteochondromas are asymptomatic and found incidentally, they can become symptomatic during adolescence or adulthood due to mechanical irritation, nerve compression, spinal cord compression, or vascular injury. In this article, we present a case of a 14-year-old patient who experienced spontaneous hemothorax caused by bleeding from a diaphragmatic laceration incurred by a costal exostosis on the right eighth rib. A preoperative chest CT scan revealed a bony projection from the rib and bloody effusion in the thoracic cavity, highlighting the possibility of bloody pleural effusion due to costal exostosis. It is important to note that costal osteochondromas are a rare cause of thoracic injury and can lead to laceration of the lung, diaphragm, and/or pericardium. Surgical intervention should be considered for symptomatic rib osteochondroma, and we advocate for prophylactic surgical removal of intrathoracic exostosis even in asymptomatic patients, in order to prevent potential complications.

## Introduction

Osteochondroma is one of the most common benign bone tumors, characterized by the appearance of a bony prominence on the outer surface of the bone [1]. Although it is usually asymptomatic in most cases, clinical symptoms may vary depending on its location and size [1-3]. Despite the relative frequency of osteochondromas, their occurrence in the ribs and their association with serious complications such as hemothorax are extremely rare, even in the pediatric population [1]. Through this clinical case, we aim to present the case of a 14-year-old patient who presented to the emergency department with chest pain and subsequently developed a severe complication that required urgent surgical treatment. In addition, we highlight the importance of radiological evaluation for the diagnosis of rib osteochondromas and associated complications.

## Case presentation

**Figure 1:**
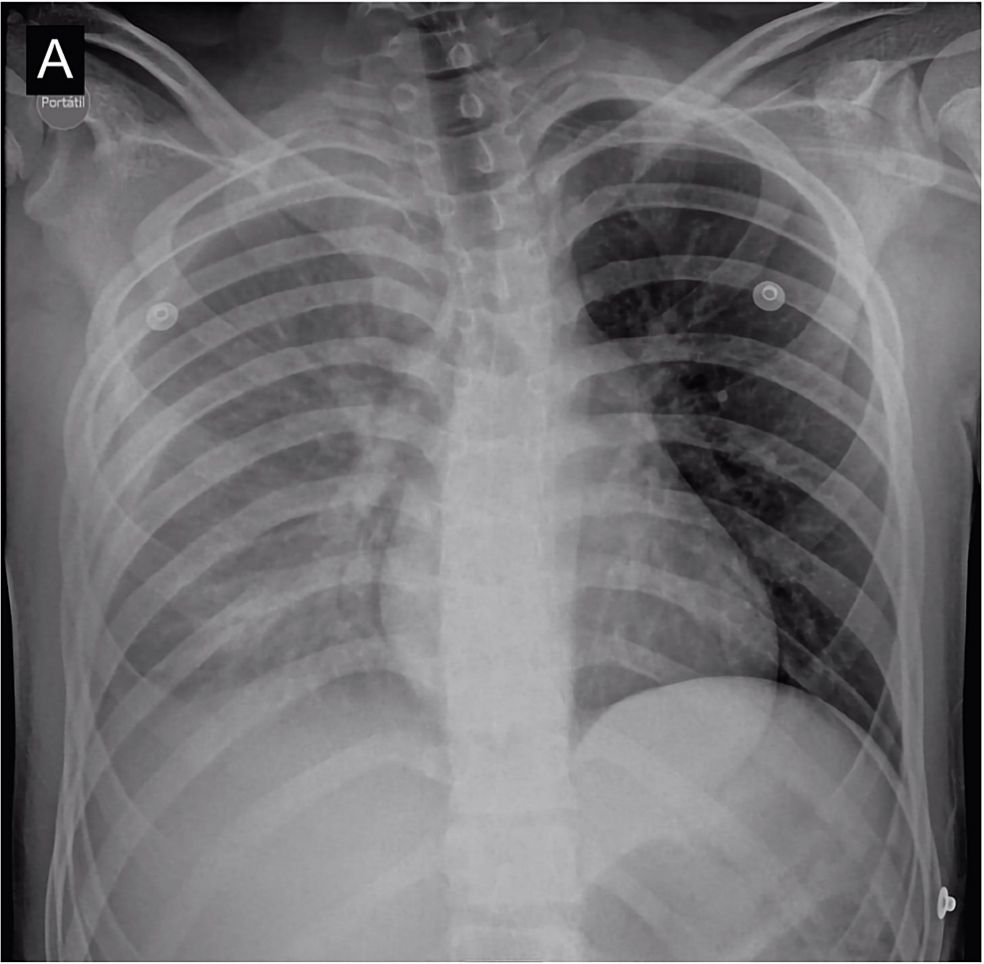
Anteroposterior chest x-ray, shows diffuse increase in the opacity of the right hemithorax due to pleural effusion.

**Figure 2:**
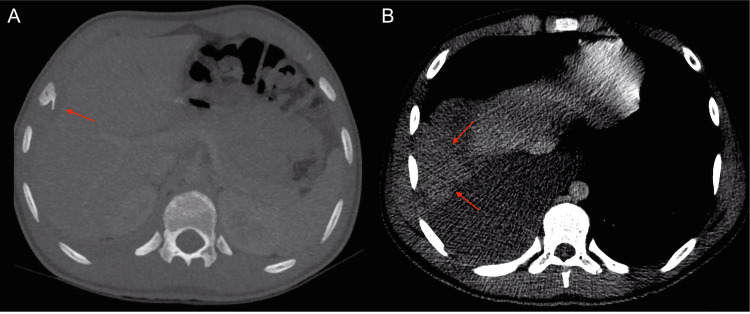
Axial chest CT in bone window (A) revealed an osseous exostosis of the inner aspect of the eighth right costal arch, which contacts and deforms the adjacent hepatic contour (red arrows). In the mediastinal view (B), a high-density pleural effusion (60 HU) was observed (red arrow).

**Figure 3:**
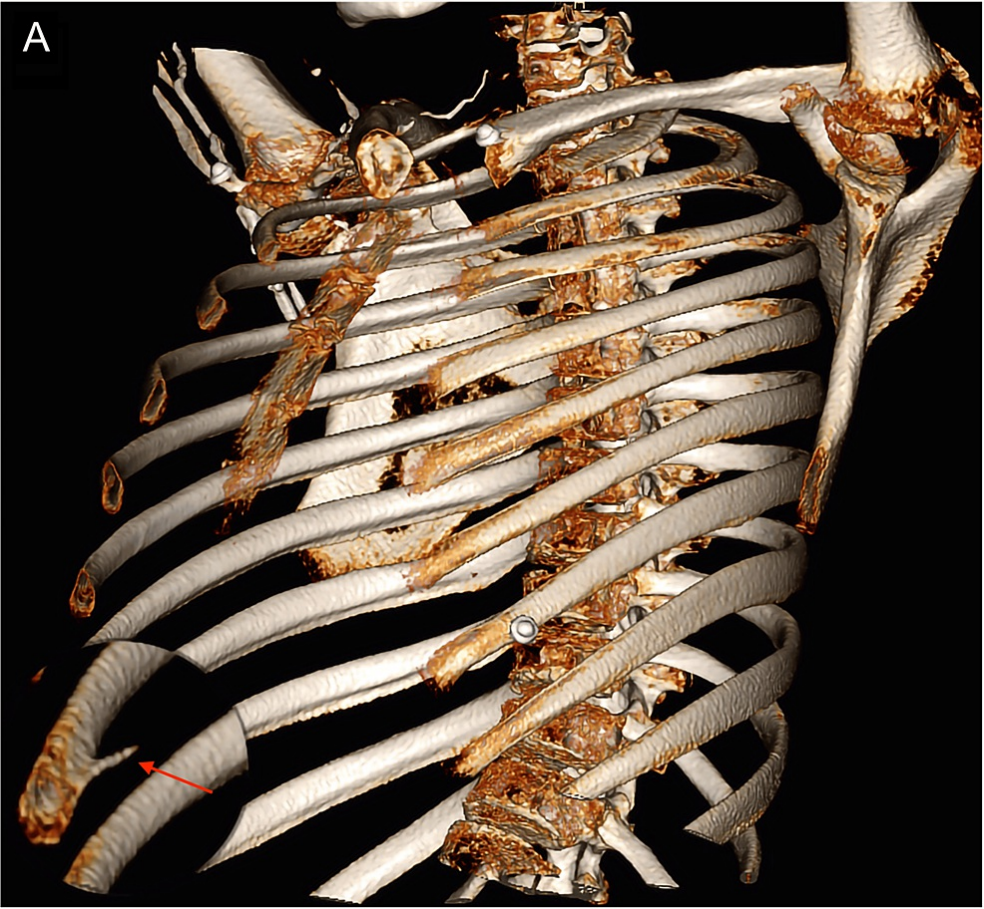
Three-dimensional computed tomography reconstruction showing the osteochondroma involving the inner aspect of the eighth costal arch (red arrow and zoom in on area of interest).

A 14-year-old patient presented with a two-week history of right-sided chest pain. The home medical evaluation suggested costochondritis. Within 24 hours, the patient experienced severe chest pain accompanied by two syncopal episodes. Upon admission to the emergency room, an ultrasound and chest x-ray revealed a right pleural effusion (Figure 1). Subsequent chest CT and three-dimensional reconstruction disclosed a bony exostosis on the inner surface of the anterior and lateral aspect of the eighth costal arch, closely associated with a high-density pleural effusion (60 UH), suggesting hemothorax. (Figures 2, 3). A closed thoracostomy was conducted, draining 500 cc of serosanguinolent fluid. Subsequently, the patient underwent thoracoscopy for resection, and histological examination ruled out the presence of a malignant lesion, confirming the diagnosis of osteochondroma. A follow-up was conducted with a chest x-ray, which demonstrated complete resolution of the hemothorax. At the one-month follow-up, the patient exhibited significant clinical improvement, with no complications related to the surgical procedure, and reported only mild discomfort at the thoracoscopy site.

## Discussion

Osteochondroma is the most common benign bone tumor [2]. Most cases manifest as solitary lesions (85%), with approximately 15% exhibiting multiple tumors in the context of multiple hereditary exostoses (HME), an uncommon genetic disorder with an autosomal dominant inheritance pattern [2]. Osteochondromas are projections or exostoses on the external surface of the bone, with the presence of cortical and medullary continuity with the parent bone and a hyaline cartilage cap [2,3]. Typically, they originate in the metaphyseal region of long bones (distal femur, proximal tibia, and humerus), growing away from the adjacent joint [2,4]. In rare instances (less than 5% of cases), they occur in flat bones such as the sternum, scapula, ribs, and pelvis [2]. In terms of primary bone tumors originating from the ribs, fibrous dysplasia represents the most common benign cause of rib growth, followed by osteochondromas [2]. Rib osteochondromas typically develop at sites where cartilage is present, such as the costochondral junction and, less frequently, the costovertebral junction [2]. 

While osteochondromas are generally asymptomatic, slow-growing masses, they can become symptomatic during adolescence due to mechanical compression, injury to adjacent structures, bursitis, secondary fractures, or even malignant transformation [2]. Malignant transformation is more common in HME [2,5,6], with an incidence estimated between 5-35% [6]. Common sites for malignant transformation include the proximal femur, proximal humerus, scapula, and pelvis [6]. This transformation typically occurs within the cartilage cap, when it increases in thickness above 3 cm in children and 2 cm in adults, leading to the development of secondary chondrosarcomas [2,6]. Most of these secondary chondrosarcomas are low to intermediate grade [2]. 

Accurate diagnosis relies on imaging modalities [2]. Key features include cortical and marrow continuity between the lesion and the parent bone, as well as the presence of a cartilage cap [2]. Initial assessment should involve plain film radiography, where osteochondromas appear as well-defined pedunculated or sessile protrusions on the bone's outer surface [2]. Pedunculated osteochondromas typically point away from the joint [2]. Additionally, a cartilaginous matrix within the cartilage cap is often visible as rings and arcs of calcifications [2]. Cross-sectional imaging such as tomography (CT) and magnetic resonance imaging (MRI) are used to evaluate the cartilage cap, and marrow continuity with the parent bone in complex anatomical regions and assess complications [2,3]. CT scans, with 3D reconstructions, clearly described the morphology, extent, and growth pattern [3]. MRI, on the other hand, excels in depicting tumor morphology, and cartilage thickness, detecting bone or adjacent soft tissue edema (bursitis), and identifying neurovascular complications [2]. The cartilage cap typically exhibits a low signal on T1-weighted images and a high signal on T2-weighted images due to its high water content [2]. 

Costal osteochondromas are rare and more commonly occur in patients with HME [3]. They represent an infrequent cause of thoracic injury [2,4]. Due to the speculated morphology, they can injure the diaphragm, pleura, heart, and adjacent lung and cause life-threatening spontaneous pneumothorax and/or hemothorax [3]. Two etiological mechanisms are proposed; one is the shearing of the pleura and diaphragm by the sharp margins of the exostosis and the other is continuous friction due to respiratory motion that leads to chronic inflammation of the pleura [7]. Notably, rib exostoses can be challenging to visualize on standard chest x-ray films [6], making chest CT more valuable in both diagnosis and the detection of intrathoracic complications [7,8]. 

Surgical resection is recommended in cases where osteochondromas emerge after puberty, become symptomatic, exhibit rapid growth, or show signs of malignant transformation [3,8]. Thoracoscopy is the preferred method for confirming the location of rib osteochondromas, determining their extent, and planning the surgical approach [4]. Compared to thoracotomy, thoracoscopy boasts lower complication rates, quicker recovery, reduced postoperative pain, and improved cosmetic outcomes [4]. Surgical techniques include segmental resection of the rib or tumor resection only [3,4]. Surgical resection plays a crucial role in preventing complications like recurrent hemothorax, pneumothorax, and intercostal neuralgia [3].

## Conclusions

Osteochondroma is a prevalent benign bone tumor, often presenting asymptomatically and frequently discovered incidentally due to its characteristic osseous surface projections. Costal osteochondromas are rare, representing an infrequent cause of thoracic injury that can lead to life-threatening complications, including hemothorax and pneumothorax. Diagnostic imaging techniques are essential for the initial assessment of these cases, with computed tomography (CT) being the recommended imaging modality. Surgical intervention is indicated for symptomatic or high-risk presentations, with thoracoscopy emerging as the preferred approach.
